# Quantitative Contact-Less Estimation of Energy Expenditure from Video and 3D Imagery

**DOI:** 10.3390/s18082435

**Published:** 2018-07-26

**Authors:** Gregor Koporec, Goran Vučković, Radoje Milić, Janez Perš

**Affiliations:** 1 Faculty of Electrical Engineering, University of Ljubljana, Tržaška Cesta 25, SI-1000 Ljubljana, Slovenia; gregor.koporec@gmail.com; 2 Faculty of Sport, University of Ljubljana, Gortanova 22, SI-1000 Ljubljana, Slovenia; goran.vuckovic@fsp.uni-lj.si (G.V.); radoje.milic@fsp.uni-lj.si (R.M.)

**Keywords:** physical activity, energy expenditure, heart rate, optical flow, scene flow, support vector machine, RBF kernel, KCF tracker, Microsoft Kinect, time-of-flight sensor, squash

## Abstract

Measurement of energy expenditure is an important tool in sport science and medicine, especially when trying to estimate the extent and intensity of physical activity. However, most approaches still rely on sensors or markers, placed directly on the body. In this paper, we present a novel approach using a fully contact-less, fully automatic method, that relies on computer vision algorithms and widely available and inexpensive imaging sensors. We rely on the estimation of the optical and scene flow to calculate Histograms of Oriented Optical Flow (HOOF) descriptors, which we subsequently augment with the Histograms of Absolute Flow Amplitude (HAFA). Descriptors are fed into regression model, which allows us to estimate energy consumption, and to a lesser extent, the heart rate. Our method has been tested both in lab environment and in realistic conditions of a sport match. Results confirm that these energy expenditures could be derived from purely contact-less observations. The proposed method can be used with different modalities, including near infrared imagery, which extends its future potential.

## 1. Introduction and Motivation

**Physical Activity** (body movement caused by muscles [1]) is both an important factor in human health [2,3] and the essential component of almost all sports. Athletes need to invest significant effort to achieve top results [1]. By measuring energy consumption, we can predict the energy requirements for individual sports activities [4,5] and detect overload. The detection of overload is an important tool for preventing muscle fatigue [6,7].

In order for the muscles to put the body in motion, their cells use energy, stored in the form of molecular bonds of adenosine triphosphate (ATP) [8]. By decomposing the ATP molecule, the cells get the necessary energy for contractions, and then again synthesize ATP by means of metabolism [8,9]. ATP decomposition and re-synthesis is a thermodynamically irreversible reaction.

**Energy expenditure** can be determined *directly* by measuring heat loss between the subject and the calorimeter in a process, called **direct calorimetry** [10]. Theoretically, after the person performs mechanical work, all of the energy in a isolated system is converted into heat, which is the basic principle on which direct calorimetry is built. Direct calorimeters are extremely expensive and impractical [10]. Energy expenditure can be also measured *indirectly* via the oxygen consumption (${VO}_{2}$) [8]. Measuring devices for so-called **indirect calorimetry** are mostly mask-like devices that must be fixed to the head of the individual [10]. Since they impair the movement, they are not suitable for routine use or even the use outside of laboratory testing. To address this problem, many **non-calorimetric methods** have been developed. They work via observing kinematics and other physiological parameters involved in the physical activity [10]. The methods in this category include measurements of heart rate, electromyography, pedometers and accelerometers, and non-contact methods.

The heart rate is inexpensive to measure and is often used as a proxy for energy consumption by amateur athletes. However, it strongly depends on physiological parameters, such as gender, height, weight, physical fitness [11] and stress levels [12]. The correct estimation of energy expenditure from heart rate needs to take all these factors into the account, and therefore heart rate, although widely used, is a poor proxy [11] for energy expenditure. Pedometers and accelerometers are the most used tools for prediction of energy consumption from kinematics observations [10]. Pedometers detect movement, associated with each step, but they are bad predictors of energy expenditure, since they can not determine the length of the step. In theory, the measurement using accelerometers can be fairly accurate, since the acceleration is proportional to the external forces [13]. They can be used both for laboratory and field research [14]. However, as noted by Zhang et al. [15], their accuracy is questionable in practice, which is consistent with later studies on fitness trackers [16]. An example of a study using contact methods is found in [17], where the authors obtained energy consumption using regression models.

Limitations of contact sensors are the main motivation behind the development of the non-contact methods. They are mostly based on the analysis of video [4,5,18,19,20]. The role of computer vision in these approaches is mainly spatiotemporal localization of activity, sometimes coupled with metabolic models, however none of them use dense estimation of motion (e.g., the motion field).

### Overview of Our Approach

**Figure 1:**
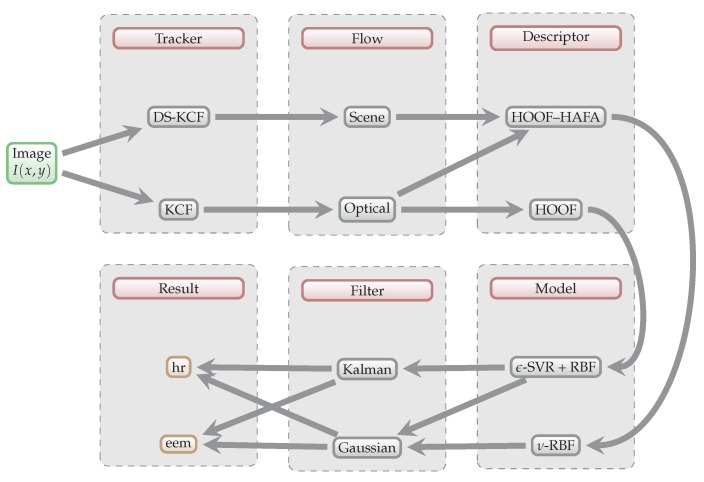
Schematic overview of our approach. Individual algorithms and methods are explained in detail in Section 3.

The key requirement of our approach is the ability to measure energy expenditure in a fully *non-contact* and *non-intrusive* manner. Non-contact methods have an obvious appeal in sport science [21]—they do not restrict the movement of athletes in any way, and therefore do not influence the results. This way, the method could be used at the matches of any competition level. Figure 1 shows the schematic overview of our approach.

In a computer vision-based system, the *motion field* is the obvious solution for measuring the intensity of physical activity, because it corresponds to kinematic movements. Motion field cannot be directly measured, but we can get its approximation via the calculation of the *optical flow*, a vector field that describes *apparent* motion of image pixels in the image coordinate system.

Finally, to estimate actual energy expenditure from optical flow, a robust descriptor of flow and the trainable model of energy expenditure are needed.

The concept of optical flow can be extended to 3D space by substituting the optical flow with the so-called *scene flow* [22]. The latter improves the measurement accuracy, because it provides the flow vectors in the real-world coordinate system. Descriptors used to describe optical flow can also be expanded for use with the scene flow.

In this paper, we present a comprehensive study on means to extract energy consumption from 2D or 3D image data, without *assuming or detecting body pose*. We examined multiple modalities of input data (RGB, near infrared and time-of-flight), different camera poses, different video acquisition technologies (IP cameras, Raspberry Pi embedded platform, and Microsoft Kinect for Windows V2) and different combinations of processing pipeline elements (HOOF and HAFA descriptors, tracking, filtering, and smoothing). Experiments have been performed both in the laboratory and in the field—during squash matches.

The paper is structured as follows: In Section 3, we present the methods and algorithms that we used to extract measurement energy consumption from image and 3D data via optical flow and scene flow, respectively. Section 4 describes the experiments and provides experimental results, obtained in laboratory environment and on the squash court. Results are discussed in Section 5, and the paper is concluded in Section 6.

## 2. Related Work

Peker et al. [19] argued that the intensity of physical activity is a subjective measurement. When observing motion in videos, each person will notice a different intensity. On this basis, the implementation of a psycho-physical protocol was presented to compare measurements of the intensity of physical activity with the help of a subjective reference. The physiology was completely excluded.

Silva et al. [18] evaluated an automatic video analysis system. By predicting types of physical activity, they aimed to demonstrate that certain computer vision methods, such as off-line image segmentation, player detection and player tracking using Kalman filter are also suitable for physical activity estimation. Unlike [19], objective methods are used. Subject velocity is used to estimate intensity of physical activity, but it yields only a rough estimate. The method has not been compared to any of the established energy expenditure measurements methods, e.g., indirect calorimetry.

Osgnach et al. [5] showed that it is possible to evaluate energy demand of football using video analysis. They employed a physical-physiological model, which can define energy consumption in terms of velocity and acceleration. They found that energy consumption at different velocities is similar, since it depends far more on acceleration and deceleration. Acyclic activities such as jumping, kicking, etc. were not taken into the account, which is a limitation of their model—it cannot be used for a range of other sports and activities. The work does not include any comparison to a reliable ground truth for energy consumption.

In ref. [4], researchers developed a method of estimating energy consumption using metabolic models of fundamental activities (walking, running, sitting, racket strikes and serving) for use in analysis of a tennis matches. Metabolic models were obtained by measuring oxygen consumption using K4B2 ergospirometer (Cosmed, Rome, Italy). Duration of individual fundamental activities was determined by video analysis of the match. These data were then used to calculate energy expenditure. By analyzing 16 games, the correlation coefficient was CORR=0.93, proving that the method quite accurately determines the energy consumption for each type of activity. Validation of models was performed by employing indirect calorimetry. Nevertheless, the development of metabolic models is not trivial and requires considerable time and expertise. The developed models are limited to a specific type of sport.

Nathan et al. [20] tried to estimate the energy consumption with Microsoft Xbox Kinect V1 sensor (Microsoft, Redmond, USA). The device was used to record motion of the skeleton. Energy consumption was determined using Gaussian regression models. For the ground truth, the Cortex Metamax 3B automated gas analysis system was used (indirect calorimety). The concordance correlation coefficient for this approach was CCC=0.879. RMS error was 8.384 kJ (2.004 kcal). Authors found that, using their method, they can easily estimate energy consumption only for high-intensity activities such as jumping. This limits the usefulness of such a method. The clear limitation of the method is the requirement for skeleton model and fitting (joint tracking). Unlike previous mentioned works based on physical and metabolic models [4,5], this method can be applied to various sports, and is most similar to approach presented in this paper.

The advantage of our method in comparison to [20] is that it does not require skeleton modeling and joint tracking. Joint tracking reliably works only on 3D data, but our method works either with 3D or 2D data, simplifying the overall setup.

## 3. Algorithms and Methods

We assume that the energy consumption in human body *due to body movement dominates all other causes*. Therefore, this component of energy consumption can be derived by observing the kinematics [10] of human body, if all other causes of energy consumption are considered noise. To arrive to the estimate of energy consumption, we used a processing pipeline with several components. The source code for parts of the processing pipeline is available on GitHub as described in the section “Supplementary Materials”.

### 3.1. Optical Flow

**Figure 2:**
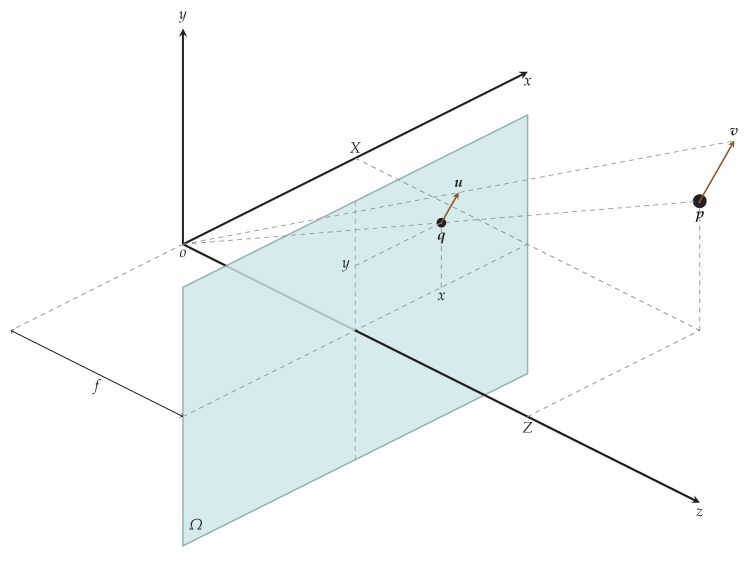
Projection of velocity field H onto image plane Ω results in optical flow O. In the camera coordinate system, a particle p with velocity field vector v∈H has an image q with motion field vector u∈G on an image plane Ω. In reality, we can only get an approximation to motion field vector u which is optical flow vector w∈O.

In theory, any mechanical movement can be described by the velocity field H . Motion field G can be understood as a projection of H onto the image plane, as shown in Figure 2. In practice, we cannot measure the motion field in a non-contact way, so we use approximations [23]. Optical flow O is a good approximation of motion field at the points of a high contrast and constant illumination.

We decided to use Farnebäack algorithm [24] for *dense* optical flow calculation—optical flow is estimated for each pixel in the image. If we know the position of person in image coordinates, and in absence of occlusions, we can get an approximation of motion field for the whole body. We used the Farnebäack algorithm implementation from the OpenCV 3.1.0 library with the following parameters: pyramid scale pyr_scale=0.5, number of pyramid layers levels=3, averaging window size winsize=15, number of iterations on each pyramid layer iterations=3, size of pixel neighbourhood poly_n=5 and standard deviation of the Gaussian poly_sigma=1.2.

### 3.2. Scene Flow

Optical flow O represents the approximation of motion field G, which is the projection of velocity field H to the image plane Ω [23]. Looking from a different perspective, optical flow is the projection of motion field approximation M. By analogy, it can be called *Scene Flow* [25]. It is a better estimation of mechanical motion than optical flow, but additional information is needed to extract it from the image data. For this purpose, we used Time-of-flight (ToF) camera, built into the inexpensive Microsoft Kinect for Windows V2 sensor [26] (Microsoft, Redmond, USA). This sensor yields registered RGB-D (red, green, blue and depth) data. To obtain scene flow, we used the PD-Flow algorithm, which has publicly available implementation [27].

### 3.3. Flow Descriptors

Raw optical flow and raw scene flow are still high-dimensional representations of scene motion. They contain noise due to violation of assumptions about motion continuity and constant illumination, and, additionally, due to inherent noise of imaging sensors [28]. It should also be noted that the number of pixels representing the observed person varies over time as the distance between camera and the person changes. Therefore, a compact and robust representation of flow that will reduce the influence of noise is needed [29].

#### 3.3.1. Histograms of Oriented Optical Flow

Chaudhry et al. [29] suggests the use of histograms of oriented optical flow (HOOF), which are invariant to scale and direction of motion. Each optical flow vector assigned a bin, relative to its angle, and weighted by its length.

Optical flow vector $w={\left[w_{x}\;w_{y}\right]}^{⊤}$ has an amplitude (Equation (1)) and a direction (Equation (2)) which is defined on an interval (Equation (3)) [29].

(1) $$\begin{array}{cc} ∥w∥= & \sqrt{w_{x}^{2}+w_{y}^{2}} \end{array}$$

(2) $$\begin{array}{cc} \Theta = & \tan^{-1}\left(\frac{w_{y}}{w_{x}}\right) \end{array}$$

(3) $$-\frac{\pi }{2}+\pi \frac{b-1}{N_{HOOF}}\leq \Theta <-\frac{\pi }{2}+\pi \frac{b}{N_{HOOF}}$$

**Figure 3:**
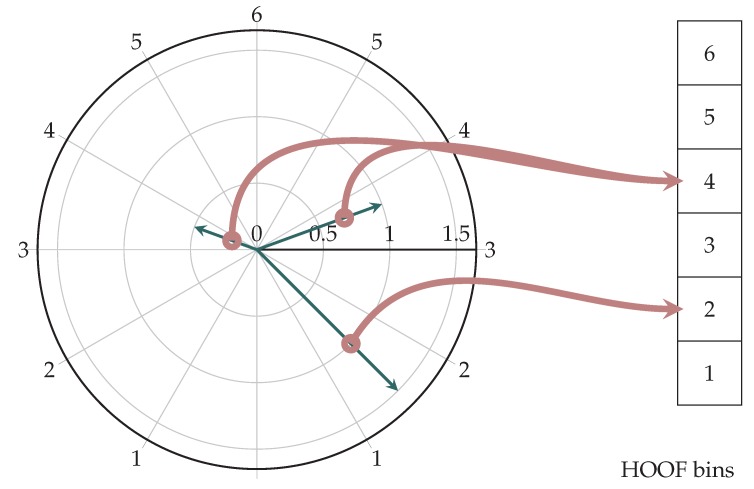
Example: Calculating a six-bin HOOF histogram. Note the invariance in the direction along the *x* axis—this is intentional.

$b,\;1\leq b\leq N_{HOOF}$ is histogram bin, where $N_{HOOF}$ is total number of histogram bins. Equation (3) indicates that vectors w are mapped from interval $\left(\frac{\pi }{2},\;\frac{3\pi }{2}\right)$ to $\left[\frac{-\pi }{2},\;\frac{\pi }{2}\right]$. The latter interval is then divided to $N_{HOOF}$ bins. Each vector w that lies in the subinterval of *b* will contribute its length ∥w∥ to bin [29]. The resulting histogram is normalized, so its sum is equal to 1. The example is shown in Figure 3.

This approach results in a histogram that is invariant to the direction of motion along the *x* axis [29], as there is no reason leftward motion would result in different energy expenditure than rightward motion. By normalizing the histogram, we get scale invariance [29] as well. Since each contribution of a vector is proportional to its amplitude, noise vectors do not influence the histogram shape [29]. Consequently, a histogram for the entire image can be determined. The only parameter that needs to be defined is $N_{HOOF}$. In our research, we determined the optimal value of $N_{HOOF}=60$.

#### 3.3.2. Histograms of Absolute Flow Amplitudes

The HOOF descriptor models only the direction of motion, and therefore contains only part of the information needed to estimate energy expenditure, the rest being contained in the intensity of motion. The latter is best described by flow vector amplitude. The idea for modeling the amplitude was adopted from [30], where the authors used histograms of optical flow (HOF) to describe motion.

**Figure 4:**
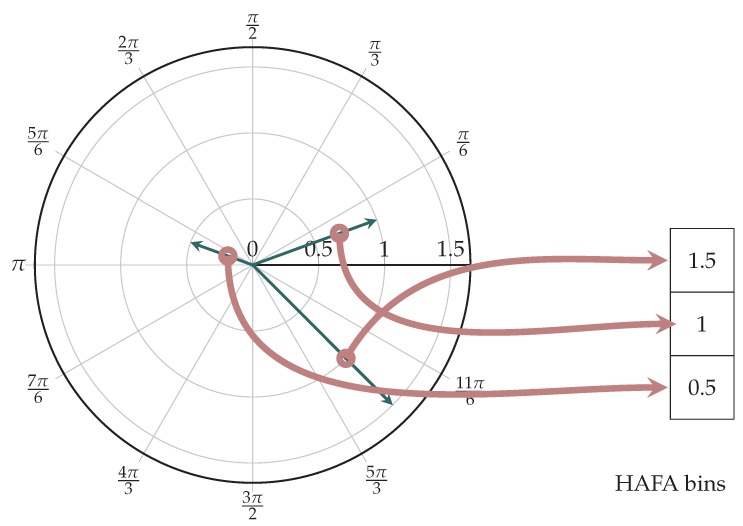
Example: Calculating a three-bin HAFA histogram.

Optical flow vector w has an amplitude defined in the interval [0,∞), which can be quantized by Equation (4). Amplitude values above the maximum are saturated and end up in the last bin. This means that amplitude ∥w∥ is defined by bin $b,\;1\leq b\leq N_{HAFA}$, where $N_{HAFA}$ is the total number of histogram bins. When such histogram is normalized, it is called the histogram of absolute flow amplitudes (HAFA). Histogram has only one parameter $N_{HAFA}$, and we established that $N_{HAFA}=60$ is appropriate value for our task and results in no human motion-caused saturation. The mapping of flow vectors to HAFA histogram is illustrated in Figure 4.

(4) $$\frac{b-1}{N_{HAFA}}\leq ∥w∥<\frac{b}{N_{HAFA}}$$

#### 3.3.3. Extension of Histogram Descriptors for Scene Flow

HOOF and HAFA histograms can also be used for scene flow. The amplitude in Equation (1) can be replaced by the amplitude in Equation (5) for scene flow vector $\mu ={\left[\mu_{x}\;\mu_{y}\;\mu_{z}\right]}^{⊤}$.

(5) $$∥\mu ∥=\sqrt{\mu_{x}^{2}+\mu_{y}^{2}+\mu_{z}^{2}}$$

The direction Θ is determined by mapping μ to image plane Ω using Equation (6).

(6) $$\Theta =\tan^{-1}\left(\frac{∥w∥}{∥\mu ∥}\right)$$

**Figure 5:**
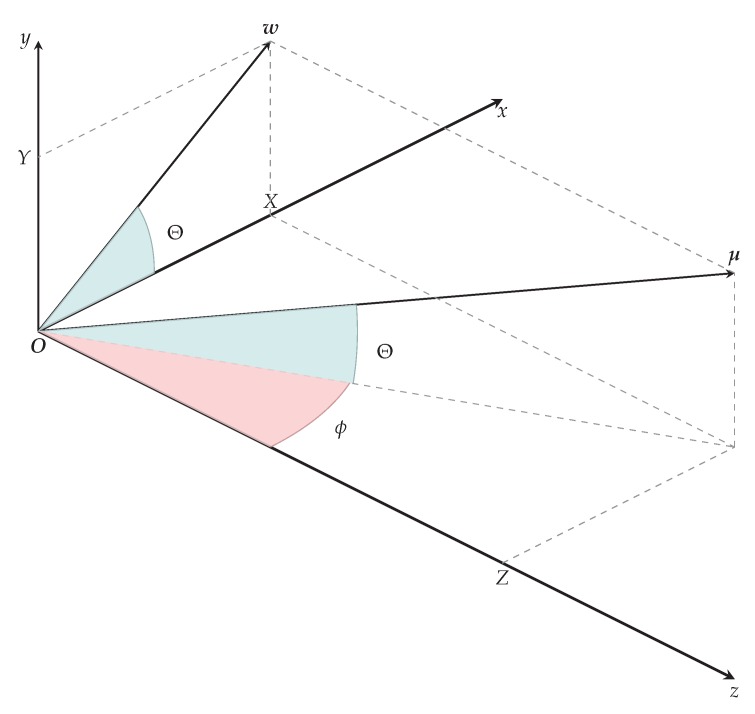
Spherical coordinates in camera coordinate system help us to extend histogram descriptors for use with the scene flow.

The equations are based on transition from Cartesian coordinates (x,y,z) to the spherical coordinates (r,ϕ,Θ). Figure 5 shows the spherical coordinates in the coordinate system of the camera. For simplicity, the image plane passes through the camera center O. To uniquely determine the space points, Θ lies in the interval $\left[-\frac{\pi }{2},\;\frac{\pi }{2}\right]$ and ϕ on the interval $\left[0,\;2\pi \right)$.

It can be seen that we lose information about the depth direction in HOOF histogram, since the azimuth angle ϕ is not taken into the account. However, this is acceptable, because the histogram must be invariant to the direction from left to right. This direction is represented by azimuth angle ϕ.

### 3.4. Mathematical Models

HOOF and HAFA descriptors are relatively low-dimensional representation of the observed motion. However, their relation to energy consumption is unknown. To solve this problem, we turn to regression modeling. In our model, we predict *instantaneous* energy consumption for *every frame of the video sequence* and only later apply Gaussian filtering to enforce temporal constraints on the output data.

Machine learning with support vectors (SVM) is often used for regression models (SVR) [31]. Their popularity is based on the high performance without the need for prior knowledge [32]. In our methods, we used ϵ-SVR and ν-SVR regression models with RBF kernel. RBF kernel is determined by Equation (7). Regression methods and RBF kernel implementations from LIBSVM library were used and are further described in [31].

(7) $$K_{RBF}\left(x_{i},x_{j}\right)=e^{-\gamma {∥\begin{array}{c} x_{i}-x_{j} \end{array}∥}^{2}}$$

### 3.5. Validation and Hyperparameter Optimization

Optimal parameters for SVM learning are not known in advance. The best way to find them is by optimization [33]. A parameter grid search approach is often used, where models with different combination of parameters are evaluated by K-fold cross-validation. For details, refer to [33].

One of the problems that plagued the initial phases of research was the over-fitting of the SVM model. Parameter ν for ν-SVR regression is described as the lower limit of the support vectors ratio in [34], and therefore cannot be used to limit the over-fitting. To solve this problem, we developed ν-RBF grid search. ν-RBF grid search is essentially the grid search described in [33] with additional constraint and filtering.

To perform ν-RBF grid search, we used a five-fold cross-validation with ν-SVR regression and RBF kernel. For every iteration *i* of the grid search, cross-validation predictions are first filtered using Gaussian filter. The filter parameter σ is equal to the value used when filtering test results. Then, we calculated mean squared error ${\bar{e}}_{MSE}^{i}$ and support vector ratio nSV by Equation (8) where $n_{SV}$ is the number of support vectors and $n_{D}$ the number of feature vectors.

(8) $$nSV=\frac{n_{SV}}{n_{D}}$$

Best iteration *i* (where we get SVM parameters) is chosen by the constraint in Equation (9), where $\nu_{max}$ is parameter of proposed method. It represents the upper limit of support vector ratio.

(9) $$nSV\leq \nu_{max}$$

### 3.6. Tracker

The position of subjects that are being observed is impossible to constrain outside of the carefully arranged laboratory setup. Note that we use flow fields without any assumption about skeleton pose, and, without any further constraints, the dominant component of the histograms would be noise. Therefore, it is necessary to introduce a tracking algorithm, to localize the position of the observed subject in the image coordinate system. Although our cameras are stationary, this is a step towards countering ego-motion of the camera as well.

When using optical flow as source of measurement, KCF tracker [35], as implemented in OpenCV 3.1.0 library, was used. For KCF tracker, we used default parameters, as specified in the OpenCV library.

When using scene flow as the source of data, we used DS-KCF tracker [36], which uses both the depth and the RGB information. Its core is based on the KCF tracker defined in [37]. The target model is represented by a feature vectors consisting of histogram of oriented gradients (HOG) of color images and a HOG of depth images.

The result of the tracking in both cases is the bounding box of the observed subject. The inside of the bounding box is used to fill the histograms in the histogram descriptors; the rest of the flow in the scene is discarded. The tracking algorithms are needed only in field tests, where the position of the player is unknown. However, in that case, they are the fragile part of our approach. If they fail to track the player, the data for that particular interval of time are missing, unless the tracking was supervised and errors corrected. The failure detection of tracking algorithms relies on the following:

- Tracking algorithm signals that it did not detect a target.
- Tracking algorithm signals low confidence in the result (mainly due to occlusions).
- Bounding box area equals zero.
- All optical flow or scene flow vectors inside bounding box are zero.

However, our approach is modular enough that the tracking algorithms can be replaced easily. With any advances in the field of visual tracking, the reliability of our method will improve.

### 3.7. Enforcing Temporal Continuity

We process the input data, either the optical flow or the scene flow, on frame by frame basis. Technically, both flows are actually velocities and directions, calculated from the comparison of each previous and current frame. Due to physical/mechanical constraints of human body, at current widely used video acquisition speeds (25–30 frames per second), the actual velocities and directions *in the observed scene* can change only for a very small amount between consecutive frame pairs.

However, due to various factors (mostly image noise), the estimations of flow and derived physiological parameters are noisy. Therefore, it makes sense to enforce temporal continuity and thus use multiple sequential measurements for better estimation of the predicted physiological parameter. For this purpose, we used Gaussian filtering. The filter was implemented using Equation (10). For the size of the kernel, we defined 3σ, where σ is standard deviation. Kernel was normalized to a sum of 1.

(10) $$g\left(x\right)=\frac{1}{\sqrt{2\pi }\sigma }e^{-\frac{x^{2}}{2\sigma^{2}}}$$

### 3.8. Extending the Algorithm to Multiple Kinect Sensors

**Figure 6:**
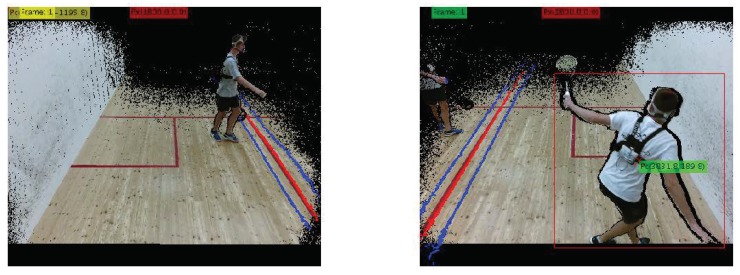
Determining the intersection of visible fields of view in left and right Kinect camera. First frames of the first set in second phase of the field experiments are shown. The fourth player is marked. The green color represents the selected camera. The intersection is a red line. Blue lines are hysteresis thresholds for switching between cameras. They lie 200 mm to the left and to the right of the intersection, shown in red.

Due to the narrow field of view of Kinect cameras, two Kinect sensors were needed to cover the entire width of the squash court, where part of the experiments took place. The time-synchronized sequences of frames were then combined with respect to the observed player before further processing, as follows:

- We obtained intrinsic parameters of infra-red (IR) sensor from Kinect camera using libfreenect2 0.2 library [38].
- The exact extrinsic parameters of the cameras were estimated manually from the intersection between fields of view of the two cameras. The intersection is shown as a red line in Figure 6.
- Using this method, we defined translational vector and rotational matrix from Euler angles.

Obtained RGB-D data from Kinect sensors was then used as follows. By tracking selected player with DS-KCF tracker, we determined the center of the target in metric units for each frame using projection matrix. If the center of the target did not contain any depth data, we selected closest point with valid depth.

The first frame of the combined sequence is the frame where the player first appears. Subsequent frames are then selected according to target center position, with the hysteresis shown as blue lines in Figure 6. The used viewpoint is changed only if the player center crosses the hysteresis line on the farther side of the court. The distance between both blue lines was set to 400 mm.

### 3.9. Evaluation

For the evaluation of the above described methodology, we built (trained) and tested many different models for heart rate and energy expenditure prediction. The training and testing data were acquired both in the laboratory and during squash matches.

Since there are many factors that may influence of the algorithms described above, we designed a batch of experiments that evaluates each factor separately, as demonstrated in the next section.

For comparison between the different models, we chose the following validation measures: correlation coefficient (CORR), relative absolute error (RAE) and root relative square error (RRSE) [39]. We also added ratio between number of support vectors and number of training data (nSV). Higher values of CORR are better, while lower values of RAE, RRSE and nSV are better.

## 4. Experiments and Results

### 4.1. Data Acquisition

Experimental data were acquired with the participation of active squash players, which are shown in Table 1 and Table 2, along with their anthropometric and physiological data. In the remainder of the paper, we refer to all of the test subjects with the abbreviation SUBJ*n*, where *n* is the unique number assigned to the subject. All subjects gave their informed consent regarding the use of the acquired data (2D and 3D video, energy consumption data and heart rate), before they participated in the data acquisition process. The study was conducted in accordance with the guidelines set by the Ethics Committee of Faculty of Sports at University of Ljubljana.

The experimental phase was carefully planned to examine many variables that may influence the results, as follows:

- *Predicted variable*: Energy expenditure eem(t) and heart rate hr(t).
- *Camera modality*: RGB (visible) and near-infrared (invisible).
- *Camera position*: Lateral and posterior view of the subject.
- *Low-level motion data*: 2D cameras (and the derived optical flow) and 3D cameras (and the derived scene flow).
- *Descriptor type*: HOOF (motion direction only) and HOOF–HAFA (motion direction and amplitude).
- *Generalization over time*: Does it matter in which phase of the game the training data are acquired?
- *Generalization over subjects*: Do the trained models work on previously unseen subjects?

It is impossible to experimentally examine the whole parameter space exhaustively (e.g., each possible combination of parameters). Therefore, the experiments have been optimized to provide the valuable insight into the performance of the proposed methods while adhering to the constraints on subject size and experiment duration. The overview of all experiments, along with experimental equipment and parameters observed, is shown in Table 3.

Accordingly, the experiments have been carried out in two different environments: in a exercise physiology laboratory (laboratory tests), and at a squash court (field tests). We addressed energy expenditure eem(t) as a central physiological parameter. Heart rate hr(t) was treated as a secondary parameter, with the full understanding that it poorly reflects the actual energy expenditure. In all cases, physiological parameters eem(t) and hr(t), were predicted from a single image of video sequence via feature vector at the time X(t). No other temporal modeling was used, except for final smoothing of predictions. This resulted in very simple, near-real-time model, which can be extended with more complex temporal modeling, should the need arise.

**Figure 7:**
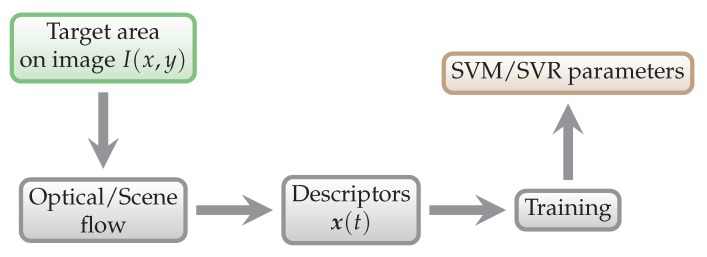
General training scheme. The data that enter this process are training data.

**Figure 8:**
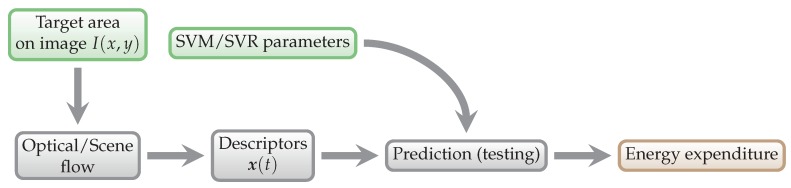
General energy expenditure prediction scheme. The data that enter this process are testing data.

The first part of the process is training of the SVM/SVR model, as shown in Figure 7. This way we obtain parameters of the regression model, which are then used to predict energy expenditure from the testing data, as shown in Figure 8.

The experiments are divided into the two main phases. In **Phase 1 experiments**, we analyzed the observability of the selected physiological parameters. We denoted the parameters *observable* if there is non-zero (positive) correlation between our estimation of a parameter and the corresponding ground truth value. In the **Phase 2 experiments**, we optimized the segments of processing pipeline for estimating physiological parameters.

### 4.2. Phase 1 Laboratory Experiments (eem(t) and hr(t), Varying Camera Angle and Camera Modality, One Subject)

The first set of experiments was performed in exercise physiology laboratory, with subject running on a treadmill in the presence of the operator—a physician, who determined the intensity and duration of workload. As part of Phase 1 test, we examined the possible use of of RGB and near-infrared (NIR) cameras, to facilitate recording in poor light conditions.

### 4.3. Data Sampling

Heart rate and energy expenditure were measured for an athlete, denoted as SUBJ0. Energy expenditure was measured using indirect calorimetry with Cosmed CPET Metabolic Cart. System allows breath-by-breath measurement [40]. We used Hans Rudolph face mask with prescribed minimal VD (dead space).

#### 4.3.1. Video Acquisition

**Figure 9:**
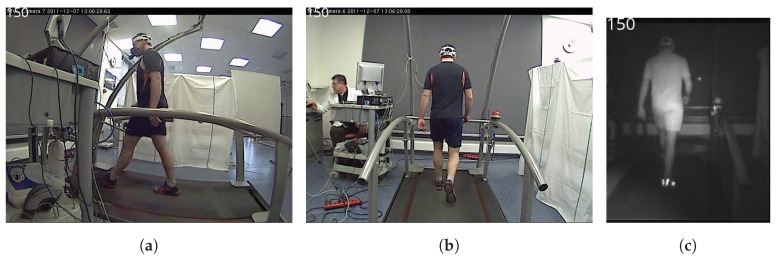
Side-view back-view and NIR images of 150th frame from Phase 1 laboratory testing: (**a**) side-view image; (**b**) back-view image; and (**c**) NIR image.

The treadmill with SUBJ0 was recorded from the two different angles: the side-view and the back-view. Videos were synchronized at the initial frame at the moments the measurements started, but certain amount of drift was inevitable due to the use of Axis 207W IP cameras (Axis Communications, Lund, Sweden). An example of a back-view, side-view and NIR image is shown in Figure 9.

Videos were acquired in 480×640 pixel resolution. The frame rate of RGB videos was 30 fps, and the frame rate of near-IR videos was 25 fps. The slope of the treadmill ranged from 1.5% to 2%.

#### 4.3.2. Measurement Protocol

Two series of recordings were made, with 20 min pause between them. Physiological parameters were sampled every 5 s. In the first series we made eight recordings, where every recording lasted for 2 min. The treadmill speed was increased by 1 km h^−1^ for every new recording. The first recording was acquired at a speed of 6 km h^−1^ and the last at a speed of 13 km h^−1^. In the second series, three recordings were made. Treadmill speeds were 7 km h^−1^, 10 km h^−1^ and 13 km h^−1^. The first set of recordings was used to acquire learning samples, and the second one to acquire testing samples.

#### 4.3.3. Processing

**Figure 10:**
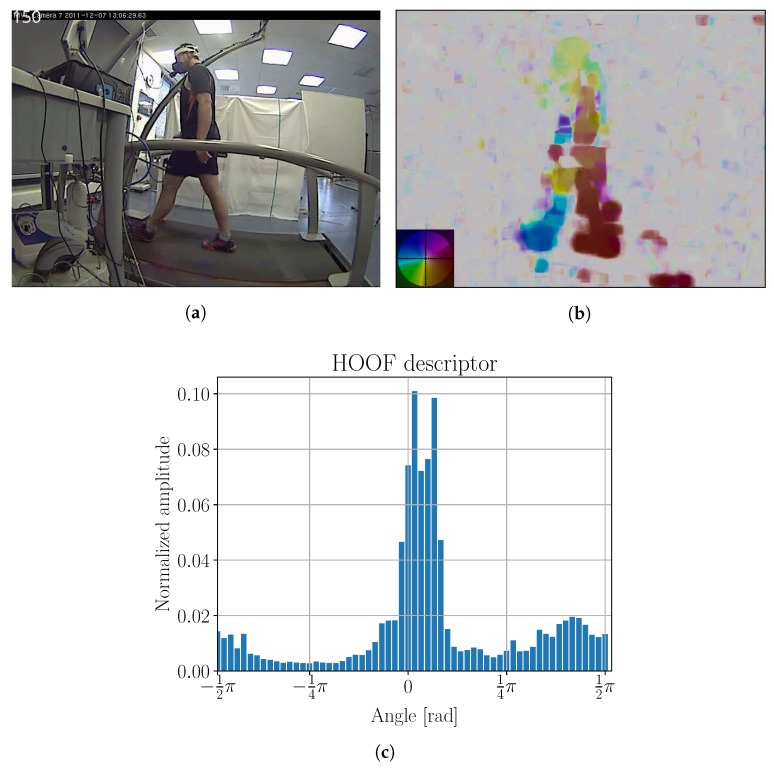
(**a**) Original image; (**b**) optical flow; and (**c**) HOOF histogram for 150th frame from Phase 1 laboratory testing. Color coding legend on the lower left in (**b**) is based on [41]. Maximal amplitude of optical flow is 17 ppf (pixels per frame).

Due to the difference in sampling rate of videos and physiological measurements, we used linear interpolation on physiological measurements to exactly align both data series. We then calculated the optical flow for the chosen area from the sequence of individual frames. An example of the obtained optical flow is shown in Figure 10b.

HOOF descriptors were calculated from the optical flow. An example of HOOF descriptor can be seen in Figure 10c.

The models have been trained using ϵ-SVR regression and RBF kernel. SVM parameters were optimized using grid search approach. We needed to determine regression penalty parameter C>0, loss function parameter ϵ>0, and kernel coefficient γ.

We have built 8 regression models, divided into the two categories: “hr” models, which predict heart rate and “eem” models, which provide the energy expenditure in kcal min^−1^. Categories are further divided according to the camera’s viewpoint: “sv” models for side-view camera and “bv” models for back-view camera.

Models were also evaluated with cross testing. This testing was done only by the type of input data (side-view or back-view). “sv” models that were made with learning samples from side-view camera were first tested with testing samples from side-view camera and then with back-view camera. Hereafter, tests with input data from side-view camera are marked with “sv” in brackets and tests with input data from back-view camera are marked with “bv” in brackets.

We also generated additional models, that we label as “mixed”. They were trained on the data both from side view and the back view. Recordings from both cameras were concatenated and cropped. This allows for evaluation of influence of the camera angle.

**Figure 11:**
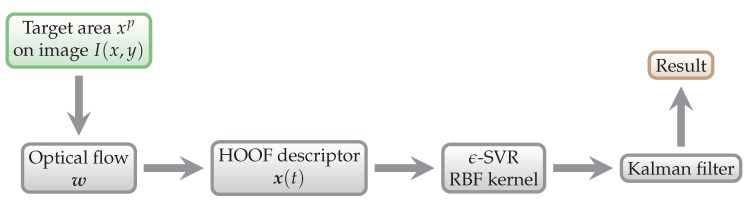
P1OFL prediction scheme. It was used for Phase 1 lab testing.

Results from all models were filtered using Kalman filter. For clearer understanding, we denote the procedure for testing all laboratory models in Phase 1 as P1OFL. It can be seen in Figure 11.

### 4.4. Phase 1 Laboratory Results (eem(t) and hr(t), Varying Camera Angle and Camera Modality, One Subject)

#### 4.4.1. Observability

**Figure 12:**
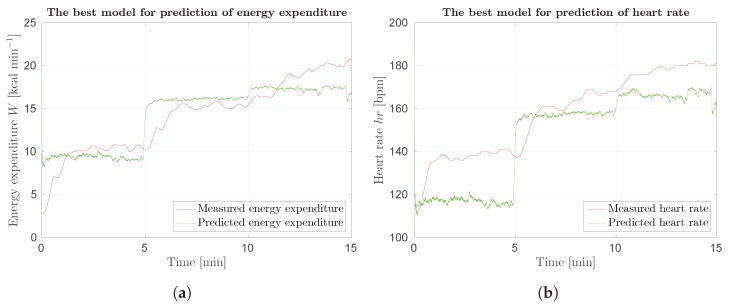
The best results for prediction of energy expenditure and heart rate. Figures show output of models eem-sv(sv) and hr-sv(sv) and the actual value of energy expenditure and heart rate: (**a**) prediction of energy expenditure; and (**b**) prediction of heart rate.

Observability results can be seen in Table 4 and Figure 12. Validation measures are average values of “sv” and “bv” models with no cross testing. Pearson correlations were averaged using Fisher *z* transform. Both energy expenditure and heart rate have strong positive correlation, which indicates that they are both observable. “hr” models are more fitted on ground truth test data, but they also have higher nSV ratio. If we look at RAE and RRSE measures, “eem” models clearly outperform “hr” models. Therefore, we can confirm that heart rate is not good physiological parameter to measure physical activity.

#### 4.4.2. Modality

Results for different viewpoint modalities with cross testing are shown in Table 5. Here, images were cropped before processing. With cropping, we manually selected target region so that it covered subject through the entire duration of the video. With this approach, we simulated ideal tracking system for better comparison with field tests.

When comparing “bv(bv)” and “sv(sv)” models, the latter are better. Worse results for back-view camera could indicate that we get less descriptive features from it. Considering cross testing (test with data from different viewpoint), we can see that all models produce poor results. CORR is very low or negative and RAE, RRSE are very high. The main difference in “mixed” models can be seen, when comparing cross tests. We can see that results, when testing models with data from different viewing angle as they were trained, are significantly better. This results indicate that better models can be obtained if we train with recordings from different viewing angles.

Results for different image type modalities are shown in Table 6. Here, images were cropped before processing. “nir” models are compared only with “bv” models, because we recorded NIR videos with only back-view viewpoint. Results for infrared sequences are better, especially for heart rate models.

### 4.5. Phase 1 Field Experiments (Squash Court, hr(t) only, Extended Descriptor)

The model squash match, consisting of only one game, was filmed in 1920×1080 resolution with RaspberryPi and RaspiCam as a recording device (Raspberry Pi Foundation, Cambridge, UK). The heart rate was measured for both players, using wearable sensors (Polar Vintage NV, Finland). First player, denoted SUBJ11, was used for training the regression model, and the second player, denoted SUBJ12, was used to test the model.

#### 4.5.1. HOOF Descriptor Extension

**Figure 13:**
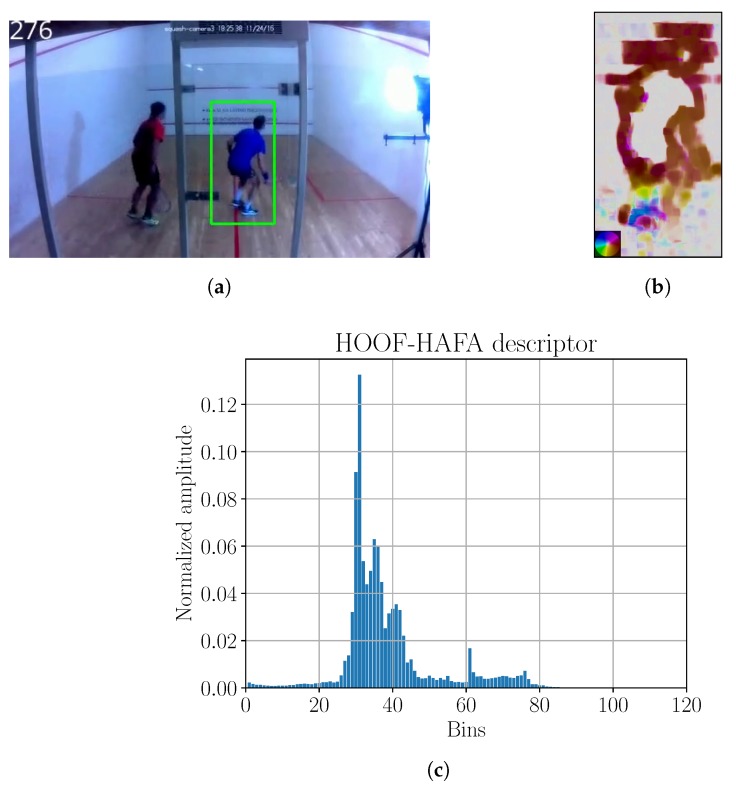
(**a**) Image with tracking; (**b**) optical flow; (**c**) HOOF–HAFA histogram. Green bounding box in sub-figure (**a**) is the detection, obtained by KCF tracker. Color coding legend on the lower left in sub-figure (**b**) is based on [41]. Maximum amplitude of optical flow image is 31 ppf.

Poor initial performance with plain HOOF descriptors in a squash game prompted an extension of HOOF descriptor with HAFA histogram to obtain an extended HOOF–HAFA descriptor. The descriptor example is visible in Figure 13c.

#### 4.5.2. Processing

Measured heart rate was linearly interpolated to obtain heart rate values at the precise timing of each frame. Next, it was filtered with a Gaussian filter with σ=16 to prevent training on overly noisy data. It was then *individualized* to each player by calculating energy expenditure based on basic equation from [42]. Predicted results from model were then converted back to heart rate of the *other player* using the same equation. This allowed us to train the model on one player, and test it on another.

To obtain player bounding boxes, tracking with KCF algorithm was employed, however the tracker was re-set once every 3 s by human operator to guarantee reasonable tracking results. We had to scale our frames to 25% of the original size, to speed up the tracking. Tracking results were remapped to the original resolution, and the example of tracking result is shown in Figure 13a.

**Figure 14:**
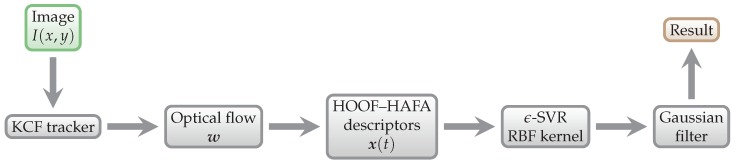
P1OFC prediction scheme, used for Phase 1 field testing.

After calculating the optical flow (Figure 13b), we used the HOOF–HAFA descriptors, where all features were scaled to the range [−1,1]. ϵ-SVR and RBF kernel were used for learning. Kalman filter was not used for squash experiments. Consistently with training, Gaussian kernel was used to filter the model output. Kernel parameter was σ=16. We denote the procedure for testing all Phase 1 field models as P1OFC. The scheme can be seen in Figure 14.

### 4.6. Normalization of HAFA Descriptors

In practice, it turns out that the original HAFA histogram used in HOOF–HAFA descriptor does not work well when the tracking is used. The target area changes its size over time, which affects the values of the HAFA histogram columns. We compensated this by introducing amplitude factor $f_{A}$.

The amplitude factor is in fact the ratio between the size of the player’s bounding box on the field tests and the target bounding box size, which, in our case, was the size of the bounding box on the treadmill. It is calculated as the ratio of the bounding box diagonals according to Equation (11), where $w_{l}$ and $h_{l}$ are width and length of the bounding box on treadmill, and $w_{s}$ and $h_{s}$ are width and length in field tests.

(11) $$f_{A}=\frac{\sqrt{w_{l}^{2}+h_{l}^{2}}}{\sqrt{w_{s}^{2}+h_{s}^{2}}}$$

### 4.7. Phase 1 Field Results (Squash Court, hr(t) only, Extended Descriptor)

**Figure 15:**
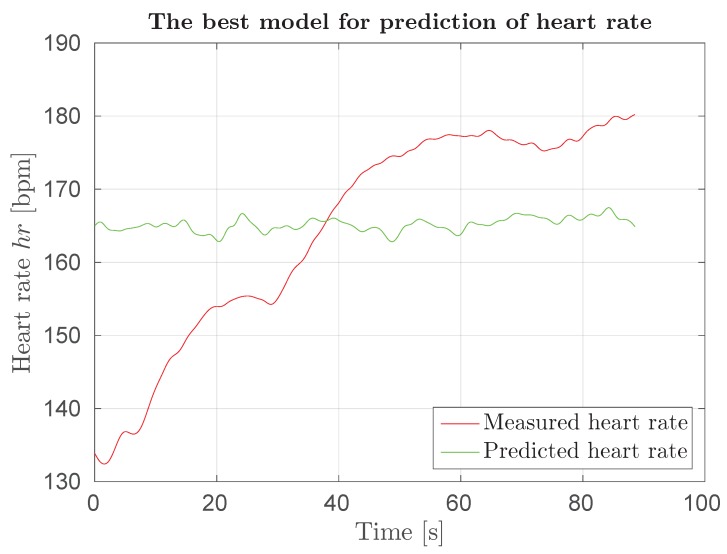
Response of model hr-bv-hoofhafa(bv) for squash game.

Field results on predicting heart rate in a squash game using different descriptors are presented in Table 7. Results show significant overfitting in one case (almost all feature vectors became support vectors) and the model with no support vectors in the other. We conclude that procedure used for laboratory testing cannot be used for field testing. Response of the overfitted model can be seen in Figure 15 and clearly confirms that such model is useless.

### 4.8. Phase 2 Laboratory Experiments (eem(t) only, 2D and 3D Data, Multiple Subjects, Generalization Test)

Seven different subjects were observed in Phase 2: SUBJ1, SUBJ2, SUBJ7, SUBJ8, and SUBJ9 (all participated in laboratory and field experiments of Phase 2). Furthermore, SUBJ4 participated only in laboratory experiments, as he was not present for the field testing. To replace him, we used SUBJ10. Between the laboratory and the field experiments of Phase 2, 43 days passed for SUBJ1 and SUBJ2, 42 days for SUBJ4 and 1 day for the others.

#### 4.8.1. Data sampling

Nowatzky stress test was carried out using a system for direct ergospirometry Cosmed K4B2 (Cosmed, Rome, Italy) on a treadmill (h/p/cosmos Sports & Medical, Nussdorf - Traunstein, Germany). This way, we obtained data energy expenditure for six different subjects (SUBJ1, SUBJ2, SUBJ4, SUBJ7, SUBJ8 and SUBJ9). Sampling frequency for energy expenditure was 0.2 Hz.

#### 4.8.2. Video and depth data acquisition

**Figure 16:**
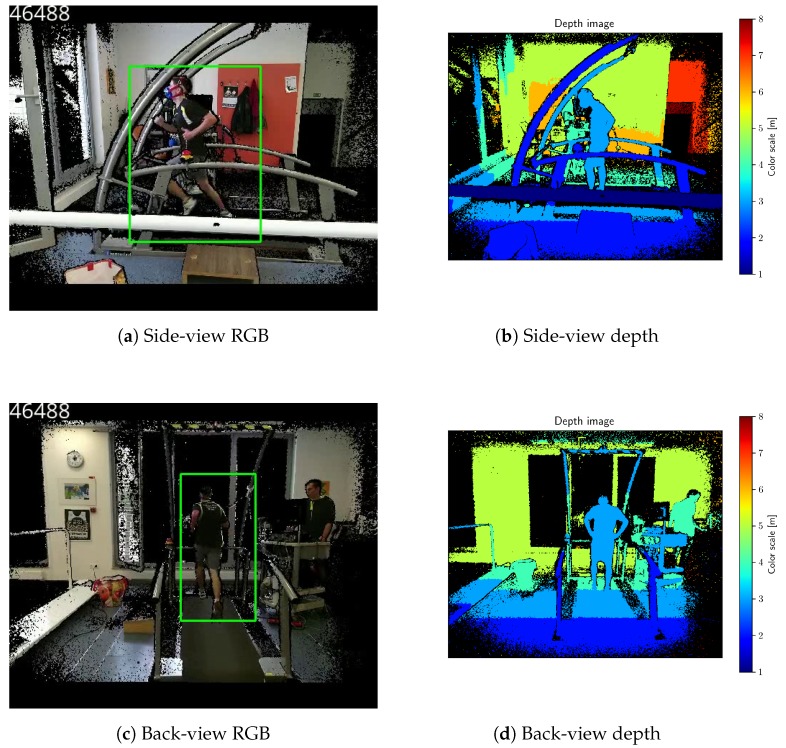
Side-view and back-view images from Kinect camera in the laboratory. RGB images were registered to corresponding depth images. Black pixels do not have corresponding depth. Green bounding boxes are target detections, provided by the KCF tracker. Treadmill speed: 16 km h^−1^.

The treadmill was recorded from the two different angles: the back-view and the side-view. Cameras were time-synchronized via the NTP protocol. When using multiple Kinect sensors, their sampling frequencies are not strictly equal. Therefore, we also obtained time stamps for each frame for the reliable synchronization. An example of a back-view and a side-view frame is shown in Figure 16.

Videos were acquired with two Microsoft Kinect for Windows V2 sensors (Microsoft, Redmond, USA) and libfreenect2 0.2 library [38]. Cameras were positioned about 2 m away from the treadmill and raised by about 1.5 m from the ground. Color (RGB) and depth images were obtained at 512×424 resolution. Image and depth sampling rate was approximately 30 fps.

#### 4.8.3. Measurement Protocol

We started the test with one minute rest on the treadmill. It was followed by a 3 min warm-up running at a speed of 5 km h^−1^ at the treadmill inclination of 0%. We continued with 3 min run at a speed of 6 km h^−1^. After 3 min, the inclination of the carpet was raised by 2% and was not changed afterwards. After the last minute of the third level (speed 6 km h^−1^, inclination 2%), the treadmill speed was increased by 1 km h^−1^ every 2 min. The test was carried out without interruption until the occurrence of objective or subjective reasons.

#### 4.8.4. Processing

**Figure 17:**
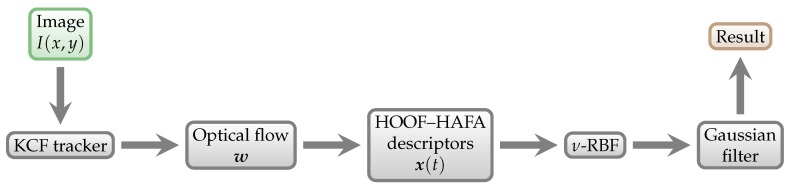
P2OF prediction scheme. It is used in for Phase 2 testing with optical flow.

Linear interpolation was used to interpolate physiological parameters. Processing was done in two different ways. The first approach, named P2OF, is based on optical flow and it is shown in Figure 17.

**Figure 18:**
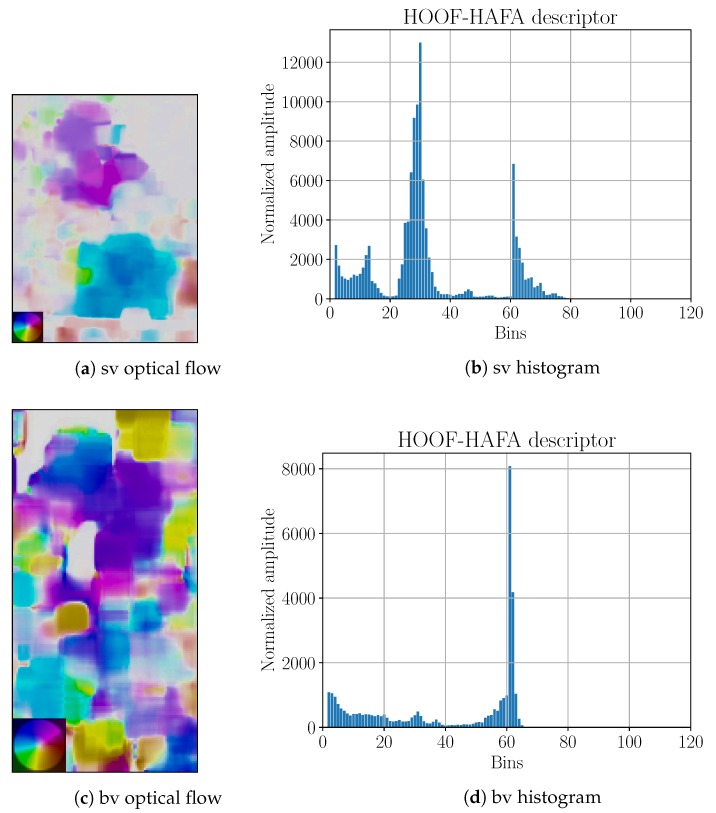
Side-view (“sv”) and back-view (“bv”) optical flows with corresponding HOOF–HAFA histograms. Optical flows correspond to 24th frame in Figure 16. Color coding legends on the lower left corners are based on [41]. Maximal amplitude for “sv” is 28 ppf and for “bv” is 5 ppf.

Bounding boxes in P2OF were obtained using the KCF tracker. The tracker was re-set once every 1 min by human operator to guarantee reasonable tracking results. Afterwards, optical flow for each frame was calculated. An example of the obtained optical flow is shown in Figure 18. Flow calculation was followed by the generation of a HOOF–HAFA descriptors with parameters $N_{HOOF}=60$ and $N_{HAFA}=60$. HAFA part of descriptors was normalized with amplitude factors $f_{A}$, which are summarized in Table 8.

**Figure 19:**
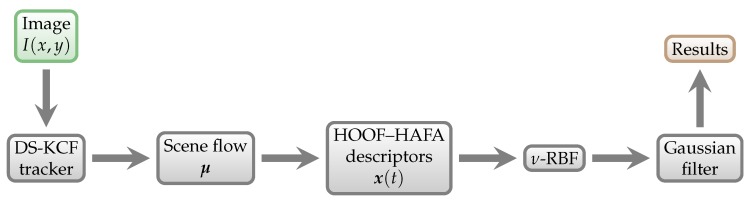
P2SF prediction scheme. It is used for Phase 2 testing using the scene flow.

The processing for scene flow is slightly different, and we denote it P2SF. Its schematic representation is shown in Figure 19.

**Figure 20:**
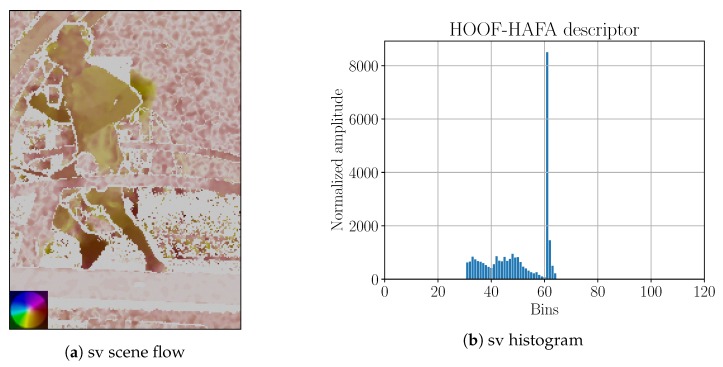

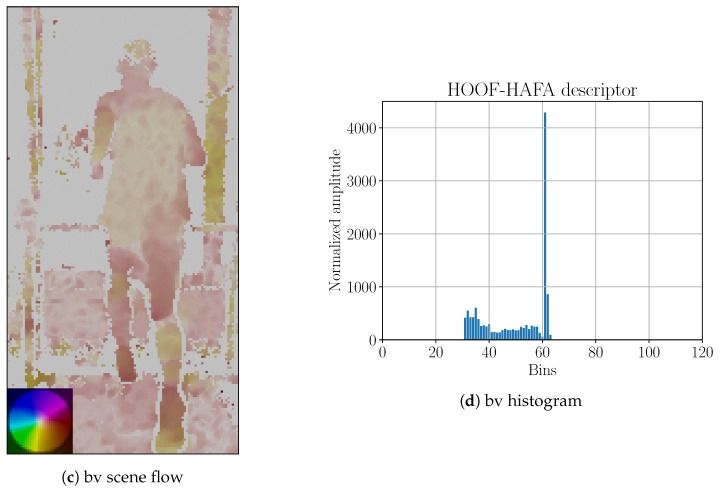
Side-view (“sv”) and back-view (“bv”) projections of scene flow onto image plane with corresponding HOOF–HAFA histograms. Scene flows correspond to the frame that is shown in Figure 16. Color coding legends on the lower left corners are based on [41]. Maximum amplitude of projected image of the scene flow: (**a**) 6 ppf; and (**c**) 15 ppf.

Bounding boxes in P2SF were obtained using DS-KCF tracker. The tracker was re-set once every 1 min by human operator to guarantee reasonable tracking results. Afterwards, scene flow for each frame was calculated. An example of the obtained scene flow is shown in Figure 20c. HOOF–HAFA descriptors were obtained as described for P2OF approach except, there was no HAFA normalization.

Models for both P2OF and P2SF approaches were trained using ν-RBF, where we used σ=5 and $\nu_{max}=0.5$. We trained two regression models for each subject: *sv* models for side-view camera and *bv* models for back-view camera. They all predict energy expenditure in kcal min^−1^.

The models were tested using three different protocols, for the following reasons:

**Protocol 1**: Every third sample of the physiological parameter is used for testing data. Others are used for model training. Results of all six subjects are averaged. This way we tried to train the model to be invariant to the fatigue in observed subjects, which increases with time.

**Protocol 2**: First 70% of samples are used for training and the remaining ones for testing. Results of all six subjects are averaged.

**Protocol 3**: We use Protocol 1, where we train models on first four subjects and test on other two. This way, we examined the possibility of generalization of the trained models to the subjects that were not part of the model training.

### 4.9. Phase 2 Laboratory Results (eem(t) only, 2D and 3D Data, Multiple Subjects, Generalization Test)

#### 4.9.1. Dependence on Fatigue Accumulation

In Table 9 are presented validation results for Protocol 1 as average of all subjects. Pearson correlations were averaged using Fisher *z* transform. All models have strong correlation with ground truth. Errors are small. We can see that scene flow models give as generally better results.

To check for the influence of subject fatigue, we tested the same data using Protocol 2. In Table 10, we present validation results for Protocol 2 as average of all subjects. Average validation measures for Protocol 1 of Phase 2 laboratory results. Pearson correlations were averaged using Fisher *z* transform. All models have poor correlation and high errors, which indicates that player fatigue alters the relation between observed motion and the actual energy expenditure. This temporal component must be taken into account when training the models—training data have to include whole duration of the match.

#### 4.9.2. Model Generalization

Using Protocol 3, we checked whether the models allow for sufficient generalization to predict energy expenditure on the subjects that were not part of the training. Results are shown in Table 11. Surprisingly, we get highest correlations for optical flow models, and not for scene flow, as expected, but error measures indicate that optical flow models are not very good. Nevertheless, the prediction results on the two subjects, SUBJ8 and SUBJ9, which were not part of the classifier training are good, which indicates that model generalizes well.

**Figure 21:**
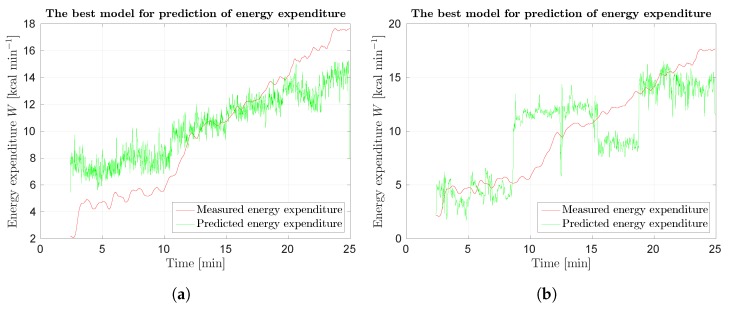
Response of SUBJ8 models for Protocol 3 of Phase 2 laboratory results: (**a**) best results using optical flow.; and (**b**) best results using scene flow. Red curve represents measured data, green curve prediction.

Best results for optical and scene flow are presented in Figure 21. Predictions are noisy, therefore wider Gaussian kernel could be used. Predictions are worse for higher energy expenditure.

### 4.10. Phase 2 Field Experiments (Squash Court, eem(t) only, 2D and 3D Data, Multiple Subjects, Generalization Test)

**Figure 22:**
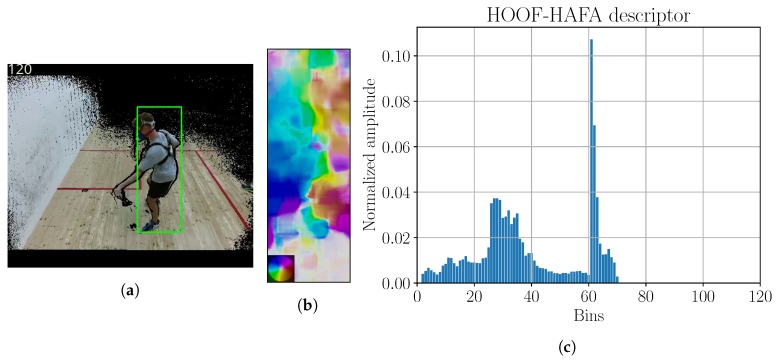

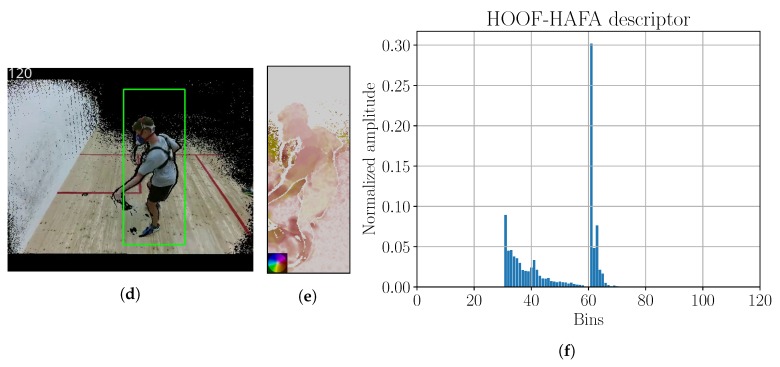
Tracking results, flow field and HOOF–HAFA histogram for P2OF approach are shown in (**a**–**c**), respectively. Panels (**d**–**f**) represent results for P2SF. Color coding legend on the lower left in (**b**,**e**) are based on [41]. Maximal amplitude of optical flow image is 10 ppf. Maximal amplitude of scene flow is 13.7 m s^−1^.

Six players (SUBJ1, SUBJ2, SUBJ7, SUBJ8, SUBJ9, and SUBJ10) played three squash matches, each containing only two games. Matches lasted 16 min, 14 min and 11 min. P2OF and P2SF processing was used, but the tracker was reset once every 3 s by the human operator. Examples of obtained tracker results, flow field and histogram descriptors are shown in Figure 22.

The physiological parameters were obtained using system for direct ergospirometry “breath by breath” Cosmed K4B2. We measured energy consumption for six different subjects with the following codes: SUBJ1, SUBJ2, SUBJ7, SUBJ8, SUBJ9 and SUBJ10.

Court was shot with two Microsoft Xbox Kinect V2 cameras using libfreenect2 0.2 library [38]. Cameras were located approximately 2 m from each other. Each camera covered half of the court. The distance from the ground was approximately 3 m, and the distance to the T-position of the squash court was approximately 4 m. Θ (rotation around the *x* axis) was approximately $30^{{}^{\circ}}$. Color (RGB) and depth (D) images were obtained at 512×424 resolution. Sampling speed was 30 fps. Cameras were synchronized using the NTP protocol. In this setup, the depth accuracy of Xbox Kinect V2 camera around the T-position is in the range of 4 mm [26].

Every game started with a 5 min warm-up. It was followed by playing two sets up to 10 points, with 2 point difference taking into account. Resting time between sets was 2 min.

### 4.11. Phase 2 Field Results (Squash Court, eem(t) only, 2D and 3D Data, Multiple Subjects, Generalization Test)

#### 4.11.1. Dependence on Fatigue Accumulation

In Table 12 are presented validation results for Protocol 1 as average of all subjects. Pearson correlations were averaged using Fisher *z* transform. We get best results for scene flow. Correlation is good, but error measures are a bit high.

In Table 13 are presented validation results for Protocol 2 as average of all subjects. Pearson correlations were averaged using Fisher *z* transform. All models have expected poor negative correlation and high errors, but they are not so different from results in Table 12.

#### 4.11.2. Model Generalization

**Figure 23:**
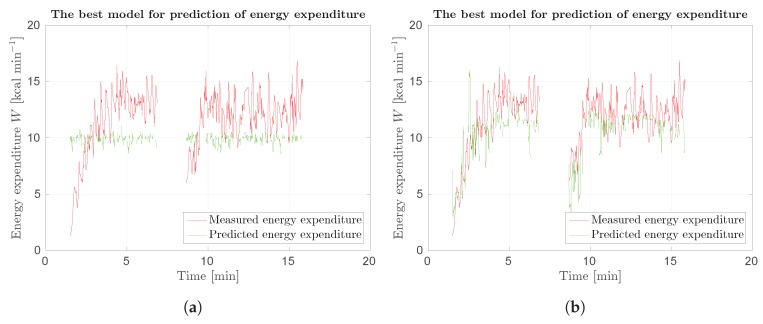
Response of SUBJ9 models for Protocol 3 of Phase 2 field results: (**a**) best results using optical flow; and (**b**) best results using scene flow. Red curve represents measured data, green curve prediction.

With Protocol 3, we wanted to check if generalized model can be used for prediction of energy expenditure on different subjects. Results are shown in Table 14. Here, we obtained the best results for scene flow, as suggested. Error measures are very high. However, as seen in Figure 23b, we get fairly good predictions. Optical flow models are also not bad, as validation results suggest. Figure 23a shows that our model predicts average value of energy expenditure.

**Figure 24:**
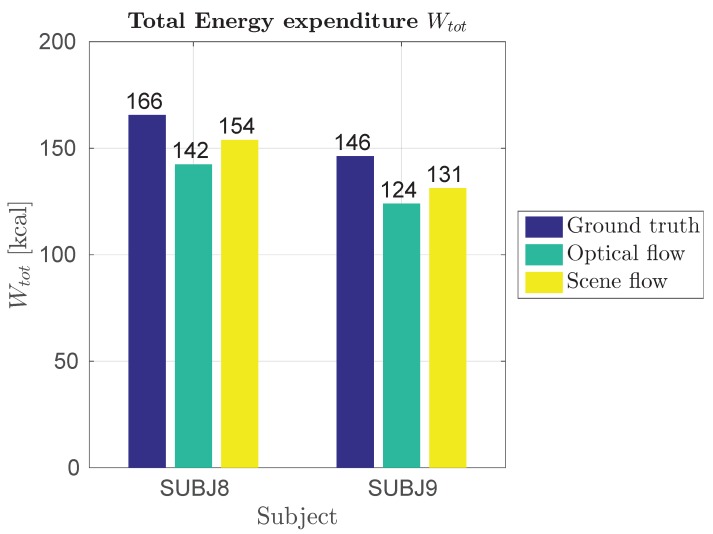
Total energy expenditure $W_{tot}$ for Protocol 3 of Phase 2 field results.

It can be concluded that generalized model using scene flow is fairly good. As we can see in Figure 24, there is small error between our model and ground truth when total energy expenditure $W_{tot}$ is taken into account.

## 5. Discussion

Experimental results have shown that the method works, under the following conditions:

- The physiological parameters we aimed to estimate are *observable* with our method, although the quality of estimation depends on the setup.
- In lab conditions, where there is no large-scale motion of subjects (only small movement of the center of mass), either HOOF or HOOF–HAFA descriptors may be used to estimate energy expenditure.
- Outside laboratory, the method has been validated only for analysis of squash matches. Due to differences in motion structure, sport-specific validation is needed for each individual sport, possibly combining the approach with large-scale motion analysis [21].
- Factors such as court size may limit the usability of currently used technology with best results (TOF camera, providing 3D data). Squash court size is on the upper limit of the size that we were able to cover that way.
- If sufficient illumination is not available, near-infrared (NIR) cameras with invisible NIR illumination may be used to acquire 2D data.
- For a model to be able to use visual data from different viewpoints, it has to be trained on data from multiple viewpoints.
- In field conditions, where subjects move across the court, tracking of the subjects (possibly amended by human intervention) is needed.
- In field conditions, the scale (apparent size) of the subject changes during the observation. Despite trying to account for these changes, 2D data (yielding optical flow) do not provide enough information to the proposed method. In this case, the scene flow, which is calculated from the 3D data, is needed, requiring either time-of-flight or stereo camera.
- Models that are to be used in field conditions have to be trained on data that span the whole duration of the match to account for changing relationship between motion and energy expenditure.
- On the other hand, properly trained models can generalize across subjects. To guarantee the performance, models used should be trained on anthropometrically similar subjects.
- Unsurprisingly, except in well controlled lab environment, the heart rate is extremely difficult to estimate from the motion data.

The proposed method compares to others as follows. For [5,18,19] we cannot compare results, because in this works subjective measure is used with no accurate ground truth using indirect calorimetry. Comparing [4] with our final generalized models in Phase 2 using scene flow, in [4] Pearson’s correlation coefficient is CORR=0.93 ours is CORR=0.995. In field testing we get CORR=0.999. Comparing [20] where concordance correlation coefficient is CCC=0.879 and RMS error is RMSE=2.004kcal we get CCC=0.989 and RMSE=9.870kcal for best laboratory model using scene flow and CCC=0.983 and RMSE=4.234kcal for best field model using scene flow in Phase 2 experiments. CCC is much higher but errors are worse. This could be corrected with higher number of support vectors. In similar approach using contact sensors [17] authors have stated results for their MCE approach (RMSE=1.192MET) and for BodyMedia, a state-of-the-art commercial EE-estimation contact device (RMSE=2.458MET). Results are for running activities. Ours is RMSE=3.262MET for best laboratory results and RMSE=2.222MET for best field result. Finally, we can perform only rough comparison with the fitness trackers. In [16], it has been estimated that the mean absolute percentage error for energy expenditure on a treadmill varied from 25.4% to 61.8% for the Fitbit Surge, from 0.4% to 26.6% for the TomTom Cardio and from 1.8% to 9.4% for the Microsoft Band. Observing Figure 24 we can estimate that our mean absolute percentage error for total energy expenditure on a squash court is 15% for measurements, based on optical flow, and 9% for those based on scene flow.

## 6. Conclusions

In this study, we explored novel contact-less methods for physiological parameters estimation from motion. To determine physiological parameters, we used optical and scene flow algorithms combined with HOOF and HAFA descriptors for robustness. SVM regression with developed ν-RBF grid search optimization was used to predict energy expenditure and heart rate of the observed subject. Tracking, Kalman and Gaussian filter were used to remove background noise and movement of objects that are not of interest. The results are comparable to other published methods, but the advantage of our method is that it is non-contact and non-intrusive, and therefore could be used at any competitive level, similarly to [21].

The proposed approach estimates the energy expenditure from the *small-scale* motion and is natural counterpart to observation of player motion on the large scale (which is essentially the motion of the center of mass) [21]. In [21], small scale motion is explicitly removed by sufficient smoothing, but, in the case of the proposed method, we use tracking to focus on the subject as related to its own coordinate system. We plan to combine both approaches for sports that include extensive motion in XY plane (e.g., soccer, basketball, and handball), to get as good an estimate of player energy expenditure as possible via completely non-contact and non-intrusive means, which is our final aim.

Nevertheless, methodology used in this paper would benefit from future work. Except for the smoothing, we do not incorporate any temporal dynamics into our model. Additionally, Kinect sensor outputs noisy depth images and there may be ways to improve (filter/smooth) them before applying our method. Combining images from multiple Kinect sensors required in our case is labor intensive. A more automatic approach for calibration of multiple Kinect sensors should be used. Better tracking algorithms for both 2D in 3D data will probably be developed in the near future and implemented in our procedure for accuracy gain.

Finally, it should be noted that our framework relies on several techniques and algorithms that were not developed by us (e.g., tracking, depth estimation, and optical and scene flow estimation), and the research in these areas is still ongoing. With advances in these algorithms, our method will become more robust, and, possibly, more accurate.

## Figures and Tables

**Table 1:** Anthropometric and physiological data for Subjects 0, 11 and 12.

Subject	SUBJ0	SUBJ11	SUBJ12
**sex**	m	m	m
**age (years)**	26	45	17
**height (cm)**	177	176	178
**weight (kg)**	79.1	68	66
${\mathrm{VO}}_{2\max}$ **(mL min${}^{-1}$)**	3705	/	/
**${\mathrm{hr}}_{\max}$ (bpm)**	194	179	203
**${\mathrm{hr}}_{\mathrm{rest}}$ (bpm)**	/	45	50
**experiments**	P1L	P1C	P1C

**Table 2:** Anthropometric and physiological data for Subjects 1, 2, 4, 7, 8, 9 and 10.

Subject	SUBJ1	SUBJ2	SUBJ4	SUBJ7	SUBJ8	SUBJ9	SUBJ10
**sex**	m	m	m	m	m	m	m
**age (years)**	20	14	15	15	19	15	16
**height (cm)**	174	151.7	186	174.4	185	175	181.5
**weight (kg)**	66.8	35.2	61.9	62.9	72.8	62	53.9
${\mathrm{VO}}_{2\max}$ **(mL min${}^{-1}$)**	3418	1908	3608	3486	3413	3513	2662
**${\mathrm{hr}}_{\max}$ (bpm)**	200	206	205	205	201	205	204
**${\mathrm{hr}}_{\mathrm{rest}}$ (bpm)**	/	/	/	/	/	/	/
**experiments**	P2L, P2C	P2L, P2C	P2L	P2L, P2C	P2L, P2C	P2L, P2C	P2C

**Table 3:** Overview of the experiments performed. Experiment names have been constructed as P1/P2 for Phase1/Phase2, L for laboratory, and C for court.

Experiment	P1L	P1C	P2L	P2C
**environment**	physiology laboratory	squash court	physiology laboratory	squash court
**equipment**	Cosmed K4B2	Polar Vintage NV	Cosmed K4B2	Cosmed K4B2
**parameter**	*eem(t)*, *hr(t)*	*hr(t)*	*eem(t)*	*eem(t)*
**camera modality**	RGB, NIR	RGB	RGB, RGBD	RGB, RGBD
**camera position**	lateral, posterior	posterior	lateral, posterior	posterior
**camera type**	Axis 207W IP camera	RaspiCam	Microsoft Kinect V2	Microsoft Kinect V2
**motion data**	optical flow	optical flow	optical, scene flow	optical, scene flow
**tracker**	/	KCF	KCF, DS-KCF	KCF, DS-KCF
**descriptor**	HOOF	HOOF–HAFA	HOOF–HAFA	HOOF–HAFA
**model**	ϵ-SVR + RBF	ϵ-SVR + RBF	ν-RBF	ν-RBF
**filter**	Kalman	Gaussian	Gaussian	Gaussian

**Table 4:** Validation measures for observability test. These are average values of *sv* and *bv* models. Pearson correlations were averaged using Fisher *z* transform. Best results shown in bold.

Model	CORR	RAE	RRSE	*n*SV
eem	0.86	**0.46**	**0.53**	**0.54**
hr	**0.89**	0.78	0.78	0.768

**Table 5:** Validation measures for viewpoint modality tests. Best results are shown in bold.

Model	CORR	RAE	RRSE	*n*SV
eem-bv(bv)	0.83	0.48	0.58	0.60
eem-bv(sv)	−0.83	1.37	1.54	0.60
eem-sv(bv)	−0.48	1.22	1.28	0.59
eem-sv(sv)	**0.86**	**0.46**	**0.52**	**0.59**
eem-mixed(bv)	0.84	0.57	0.63	0.62
eem-mixed(sv)	0.85	0.46	0.54	0.62
hr-bv(bv)	0.87	0.75	0.75	0.87
hr-bv(sv)	−0.86	2.13	2.15	0.87
hr-sv(bv)	0.33	1.08	1.22	0.85
hr-sv(sv)	0.90	0.71	0.72	0.85
hr-mixed(bv)	0.88	**0.60**	**0.62**	**0.74**
hr-mixed(sv)	**0.89**	0.67	0.68	0.74

**Table 6:** Validation measures for image type (BGR or NIR) modality tests. Best results shown in bold.

Model	CORR	RAE	RRSE	*n*SV
eem-bv(bv)	0.83	0.48	0.58	0.60
eem-nir(nir)	**0.86**	**0.47**	**0.53**	**0.58**
hr-bv(bv)	0.87	0.75	0.75	0.87
hr-nir(nir)	**0.90**	**0.67**	**0.69**	**0.73**

**Table 7:** Validation measures for field testing. HOOF and HOOF–HAFA descriptors are used. Models are overfitted.

Model	CORR	RAE	RRSE	*n*SV
hr-bv-hoof(bv)	0.00	1.45	1.46	0.00
hr-bv-hoofhafa(bv)	0.34	0.97	0.98	0.98

**Table 8:** Amplitude factors $f_{A}$ for each subject and camera viewpoint.

Viewpoint	Subject	$f_{A}$	Viewpoint	Subject	$f_{A}$
back-view	1	208.557	side-view	1	236.985
	2	179.011		2	163.957
	4	225.568		4	196.461
	7	195.133		7	205.760
	8	209.991		8	190.253
	9	182.003		9	178.16

**Table 9:** Average validation measures for Protocol 1 of Phase 2 laboratory results. Pearson correlations were averaged using Fisher *z* transform. Best results shown in bold.

Model	CORR	RAE	RRSE	*n*SV
eem-bv-of(bv)	0.97	0.35	0.38	0.33
eem-sv-of(sv)	**0.98**	**0.20**	**0.23**	**0.27**
eem-bv-sf(bv)	0.97	0.26	0.30	0.33
eem-sv-sf(sv)	**0.99**	**0.12**	**0.15**	**0.26**

**Table 10:** Average validation measures for Protocol 2 of Phase 2 laboratory results. Pearson correlations were averaged using Fisher *z* transform. Best results shown in bold.

Model	CORR	RAE	RRSE	*n*SV
eem-bv-of(bv)	**−0.51**	4.71	4.24	0.30
eem-sv-of(sv)	−0.69	**4.26**	**3.93**	**0.22**
eem-bv-sf(bv)	−0.51	4.89	4.59	0.35
eem-sv-sf(sv)	**−0.29**	**4.23**	**3.93**	**0.26**

**Table 11:** Validation measures for Protocol 3 of Phase 2 laboratory results. Best results shown in bold.

Model	CORR	RAE	RRSE	*n*SV
eem-bv-of-subj8(bv)	0.95	0.50	0.52	0.30
eem-bv-of-subj9(bv)	0.95	0.55	0.57	0.30
eem-sv-of-subj8(sv)	**0.96**	**0.34**	**0.39**	**0.18**
eem-sv-of-subj9(sv)	0.93	0.59	0.69	0.18
eem-bv-sf-subj8(bv)	**0.79**	**0.58**	**0.63**	0.33
eem-bv-sf-subj9(bv)	0.68	1.00	1.12	0.33
eem-sv-sf-subj8(sv)	0.14	1.16	1.29	**0.22**
eem-sv-sf-subj9(sv)	0.78	0.74	0.82	0.22

**Table 12:** Average validation measures for Protocol 1 of Phase 2 field results. Pearson correlations were averaged using Fisher *z* transform. Best results shown in bold.

Model	CORR	RAE	RRSE	*n*SV
eem-bv-of(bv)	0.54	0.94	0.91	0.48
eem-bv-sf(bv)	**0.76**	**0.66**	**0.65**	**0.41**

**Table 13:** Average validation measures for Protocol 2 of Phase 2 field results. Pearson correlations were averaged using Fisher *z* transform. Best results shown in bold.

Model	CORR	RAE	RRSE	*n*SV
eem-bv-of(bv)	−0.05	**1.41**	**1.34**	**0.41**
eem-bv-sf(bv)	**0.00**	1.77	1.67	0.41

**Table 14:** Validation measures for Protocol 3 of Phase 2 field results. Best results shown in bold.

Model	CORR	RAE	RRSE	*n*SV
eem-bv-of-subj8(bv)	**0.02**	**1.27**	1.17	**0.29**
eem-bv-of-subj9(bv)	0.02	1.29	**1.13**	0.29
eem-bv-sf-subj8(bv)	0.41	1.02	0.99	0.41
eem-bv-sf-subj9(bv)	**0.72**	**0.89**	**0.83**	**0.41**

## Supplementary Materials

The source code for feature extraction and tracking is available in the GitHub repositories: https://github.com/8greg8/flow-features and https://github.com/8greg8/tracker.

## Abbreviations

The following abbreviations are used in this manuscript:

3D	Three dimensional
ATP	Adenosine triphosphate
HOOF	Histogram of oriented optical flow
HAFA	Histogram of absolute flow amplitudes
SVM	Support vector machine
SVR	support vector regression
RBF	Radial basis function
KCF	Kernelized correlation filter
DS-KCF	Depth scaling kernelized correlation filter
NTP	Network time protocol
RGB	Red green blue
D	Depth
IR	Infrared
NIR	Near-infrared
CORR	Pearson correlation coefficient
CCC	Concordance correlation coefficient
RMSE	Root mean squared error
RRSE	Root relative squared error
RAE	Relative absolute error
MET	Metabolic equivalent of task
